# How perceived value, environmental awareness, and social identity shape public support for industrial heritage: the mediating role of place attachment

**DOI:** 10.3389/fpsyg.2025.1645646

**Published:** 2025-08-22

**Authors:** Yinghang Fu, Weidan Dong

**Affiliations:** Faculty of Engineering, Dongshin University, Naju, Republic of Korea

**Keywords:** environmental psychology, industrial heritage, public participation, willingness to pay, place attachment, structural equation modeling

## Abstract

**Introduction:**

Public engagement is critical to the conservation of industrial heritage sites, yet the psychological mechanisms underlying support behaviors remain understudied. This study investigates how perceived value, environmental sustainability awareness, social identity, and perceived government support shape the public’s willingness to participate in and financially support industrial heritage conservation. Particular attention is given to the mediating role of place attachment.

**Methods:**

A structured survey was administered to 385 visitors at the “*Changtuo 1958*” industrial heritage site in Changchun, China. Key constructs—including perceived value, environmental sustainability awareness, social identity, perceived government support, place attachment, willingness to participate, and willingness to pay—were measured using validated multi-item Likert scales. Data were analyzed using structural equation modeling (SEM) to test direct and indirect effects, and mediation analysis was conducted to assess the role of place attachment.

**Results:**

All four predictors—perceived value, environmental sustainability awareness, social identity, and perceived government support—significantly enhanced both participation and payment intentions. Place attachment partially mediated these relationships, with social identity exerting the strongest overall impact on both outcomes. SEM results confirmed that emotional bonds to the site act as a crucial pathway linking cognitive and social perceptions to public engagement.

**Discussion:**

These findings underscore the importance of affective and identity-based mechanisms in promoting pro-environmental behaviors toward industrial heritage conservation. Strategies to strengthen public support should include fostering emotional connections, enhancing sustainability messaging, and increasing trust in institutional support. The study provides actionable insights for policymakers and heritage managers aiming to enhance sustainable engagement in industrial heritage contexts.

## 1 Introduction

Industrial heritage sites, once centers of economic production, have increasingly gained attention as culturally and emotionally significant landscapes that embody the collective memory of communities. In the context of global deindustrialization and urban transformation, these sites are no longer seen merely as obsolete relics but as meaningful places that contribute to identity, sustainability, and a sense of belonging (Xie, 2015a; Xie, 2015b; Nocca, 2017).

Environmental psychology provides a valuable lens to explore how individuals cognitively and affectively relate to places. A central construct in this field is place attachment, which refers to the emotional bonds people form with specific physical environments (Scannell and Gifford, 2010). This concept has been widely applied in studies of pro-environmental behavior, place identity, and public participation (Ramkissoon et al., 2013; Vong, 2015). Empirical evidence suggests that individuals with strong place attachment are more likely to engage in conservation efforts, both behaviorally and financially (López-Mosquera and Sánchez, 2012; Chen and Hua, 2015).

However, industrial heritage sites present a unique psychological challenge. Unlike natural landscapes or traditional monuments, these spaces often carry complex and ambiguous meanings associated with both local pride and environmental degradation (Loures, 2015). Understanding the psychological mechanisms that shape public support for their preservation requires an integrated perspective that considers both cognitive evaluations and emotional responses.

Existing research has identified several cognitive and contextual variables that influence conservation behavior. These include individuals’ perceived value of the heritage site (Poria et al., 2013), awareness of its environmental relevance (Bullen and Love, 2010), social identity associated with the place (Murzyn-Kupisz and Działek, 2013), and perceived government support (Su and Wall, 2014). Yet, these variables are often examined in isolation, and there is limited understanding of how they interact to influence public behavioral intentions, such as willingness to participate in conservation efforts or to financially support heritage management.

Moreover, the potential mediating role of place attachment has received insufficient empirical attention. Theoretical frameworks such as the Value–Attitude–Behavior model (Stern, 2000) and the Theory of Planned Behavior (Ajzen, 1991) suggest that emotional factors like place attachment may serve as critical bridges between value-based perceptions and behavioral outcomes. Specifically, place attachment could transform abstract evaluations (e.g., sustainability concern) into tangible support behaviors.

To address these theoretical and empirical gaps, the present study poses the following key research questions:

1. How do perceived heritage value, environmental sustainability awareness, social identity, and perceived government support affect public willingness to participate in and financially support industrial heritage conservation?
2. Does place attachment mediate the relationships between these cognitive and social variables and public engagement intentions?
3. Among the four predictors—perceived heritage value, environmental sustainability awareness, social identity, and perceived government support—which exerts the strongest influence on behavioral outcomes?

To answer these questions, the study focuses on the “*Changtuo 1958*” industrial heritage park in Changchun, China a former tractor factory site that has been transformed into a multifunctional cultural district. Based on a structured survey of 385 respondents, the study employs structural equation modeling (SEM) to examine the direct and indirect effects of the four key predictors on two outcome variables: willingness to participate and willingness to pay. Particular attention is given to the mediating role of place attachment in linking perception and behavior.

By situating the analysis within the framework of environmental psychology, this research contributes to a growing literature that emphasizes the emotional, cognitive, and identity-based mechanisms underlying environmental stewardship. It also offers practical implications for policymakers and heritage managers seeking to foster public support for industrial heritage conservation in rapidly changing urban contexts.

## 2 Literature review

### 2.1 Industrial heritage conservation and sustainable development

Industrial heritage refers to cultural remains of historical, technological, social, architectural, or scientific value, including buildings, machinery, factories, mines, and processing or smelting facilities (TICCIH, 2003). Since the 1980s, the conservation of industrial heritage has gradually become an important domain within international cultural heritage preservation (Alfrey and Putnam, 1992). Xie (2015a) and Xie (2015b) emphasized that industrial heritage conservation not only involves the physical preservation of artifacts but also the transmission and sustainable use of industrial civilization. Cizler (2012) demonstrated that the adaptive reuse of industrial heritage can promote urban regeneration and sustainable development. Landorf proposed a sustainable management framework for industrial heritage, highlighting the need to balance economic, social, and environmental dimensions. Based on case studies across Europe, Loures (2015) found that successful industrial heritage projects often achieve a synergy between cultural value preservation and socio-economic benefits.

In the context of sustainable development, industrial heritage conservation faces new opportunities and challenges. Sustainability introduces new value dimensions and evaluative frameworks for heritage conservation (Auclair and Fairclough, 2015), while the Sustainable Development Goals (SDGs) further reinforce this trend by imposing stricter requirements related to environmental responsibility, resource efficiency, and social inclusion (Yung et al., 2017). Ifko (2016) stressed that industrial heritage conservation should adhere to sustainability principles, balancing preservation and development, history and modernity, and protection and innovation.

In China, the conservation of industrial heritage began relatively late but has developed rapidly in recent years (Qian, 2023). Song et al. (2023) conducted a systematic review of China’s industrial heritage conservation, identifying challenges such as limited policy support, low public participation, and financial constraints. Wang et al. (2020) explored the relationship between industrial heritage preservation and urban sustainability in China, suggesting that conservation should be integrated into broader urban renewal and regional development strategies.

### 2.2 Public participation in industrial heritage conservation

Public participation is a critical component of cultural heritage protection and is especially vital for the conservation of industrial heritage (Chirikure et al., 2010). Marta and Belso-Martínez (2020) demonstrated that effective public participation enhances the legitimacy and acceptability of heritage-related decision making. Dupagne et al. (2004) noted that public engagement fosters community identification with industrial heritage and contributes diverse perspectives and resources to conservation efforts.

A variety of factors influence public willingness to participate. Hou and Du (2020) found that individuals’ perceptions of heritage value significantly affect their willingness to engage in preservation efforts. Mydland and Grahn (2012) emphasized that social identity is a key motivator of public involvement. Veldpaus and Pereira Roders (2014) highlighted the importance of government support and institutional context in encouraging public participation in heritage conservation.

In the Chinese context, Li et al. (2019) identified cultural identity, governmental guidance, and participation mechanisms as critical determinants of public engagement in urban heritage conservation. However, systematic investigations into public participation in the context of industrial heritage remain limited. In particular, there is a research gap regarding how public awareness of environmental sustainability influences engagement in industrial heritage preservation.

### 2.3 Willingness to pay and the valuation of industrial heritage

Willingness to pay (WTP) is a key indicator for assessing the economic value of nonmarket goods such as cultural heritage (Choi et al., 2010). Throsby (2001) argued that cultural heritage embodies both use and nonuse values, and WTP provides a comprehensive measure of these. Using the contingent valuation method, Tuan and Navrud (2008) examined a Vietnamese heritage site and found that education level, income, and cultural identity significantly influenced respondents’ WTP.

In the industrial heritage domain, Lazrak et al. (2014) investigated the impact of industrial heritage on real estate values in the Netherlands, affirming its economic value. Chen and Hua (2015) applied choice experiments to assess the economic value of a Chinese industrial heritage site and revealed that public WTP was significantly affected by perceived historical value and expectations of environmental improvement. Nocca (2017) used multiple case studies to demonstrate the economic contribution of industrial heritage conservation to sustainable development.

Nonetheless, existing studies primarily focus on the valuation methods and economic implications of WTP, while psychological and social drivers remain underexplored (Báez-Montenegro et al., 2012). This study addresses this gap by examining how perceived heritage value, environmental sustainability awareness, social identity, and perceived government support influence WTP from a multidimensional perspective.

### 2.4 Place attachment and industrial heritage conservation

Place attachment refers to the emotional bond between individuals and specific locations (Low and Altman, 1992; Scannell and Gifford, 2010). Scannell and Gifford’s tripartite framework describes place attachment in terms of person (individual and collective identities), process (affective, cognitive, and behavioral components), and place (physical and social environments). Low and Altman (1992) emphasized that place attachment not only involves affinity for the physical setting but also includes social relationships and cultural meanings embedded in the place.

In the field of heritage preservation, place attachment is considered a key driver of public support and participation (Tweed and Sutherland, 2007). Vong (2015), based on research in Macau’s historic districts, confirmed that residents’ place attachment positively influenced their participation in heritage preservation.

In the context of industrial heritage, Wheeler (2014) explored community attachment in postindustrial mining regions in the UK and found that emotional bonds to industrial landscapes persisted even after economic decline. Xie (2015a) and Xie (2015b) investigated industrial heritage sites in China and suggested that place attachment facilitates the transmission of industrial memory and the reconstruction of community identity. Although earlier studies have examined the general role of place attachment in environmental behavior (e.g., Ramkissoon et al., 2013), recent research still lacks a systematic understanding of its mediating function in the context of industrial heritage conservation, especially amid China’s accelerating urban transformation (e.g., Sektani et al., 2023; Bowden et al., 2025).

## 3 Theoretical framework and research hypotheses

To synthesize the multiple theoretical perspectives adopted in this study, we conceptualize place attachment as a central affective mediating mechanism that links cognitive evaluations and social constructs with behavioral outcomes. Within the Theory of Planned Behavior (TPB), perceived heritage value and government support function as attitudinal and normative beliefs that influence intentions through emotional bonding to place. The Value-Belief-Norm (VBN) model similarly suggests that sustainability awareness shapes behavioral norms via internalized affective responses, operationalized here as place attachment. In the Social Identity Theory, individuals’ group-based identity with the heritage site contributes to place-related emotional bonds, reinforcing prosocial actions. Institutional trust, meanwhile, builds perceived legitimacy and emotional security, further deepening place attachment and motivating support behaviors. This theoretical integration underscores place attachment as a shared pathway across diverse explanatory models.

### 3.1 Perceived value of industrial heritage and public participation

The perceived value of industrial heritage refers to the public’s subjective understanding of its historical, cultural, educational, esthetic, and economic significance (Poria et al., 2013). According to the Theory of Planned Behavior (Ajzen, 1991), behavioral intentions are influenced by individuals’ evaluations of expected outcomes. When the public perceives high value in industrial heritage, they are more likely to support its protection (Hou and Du, 2020).

Research by Poria et al. (2013) shows that visitors’ perceptions of cultural heritage value significantly affect their visiting behavior and conservation attitudes. Ung and Vong (2010) found that perceived cultural value positively influences public participation in heritage preservation, based on a study of World Heritage Sites in Macau. In the context of industrial heritage, Xie and Gu (2015) demonstrated a positive relationship between the perceived historical and cultural value of industrial heritage and individuals’ willingness to participate in its protection.

The Value-Belief-Norm theory also suggests that personal values shape pro-environmental behavior (Stern, 2000). When the public recognizes the value of industrial heritage, they are more willing to invest time, energy, and financial resources into its preservation (Kang and Gretzel, 2012). Based on this, the following hypotheses are proposed:

H1a: Perceived value of industrial heritage has a significant positive effect on public participation intention.

H1b: Perceived value of industrial heritage has a significant positive effect on willingness to pay.

### 3.2 Environmental sustainability awareness and public participation

Environmental sustainability awareness refers to the public’s understanding of the relationship between industrial heritage protection and sustainable development (Bullen and Love, 2010). As environmental awareness increases, sustainability becomes a major public concern (Yung and Chan, 2012). The adaptive reuse of industrial heritage can reduce resource consumption and environmental impact, aligning with sustainable development goals (Loures and Vaz, 2018).

Bullen and Love (2010) found that the environmental benefits of adaptive reuse are a key factor in public support for historic building conservation. Yung and Chan (2012) confirmed that awareness of environmental sustainability positively influences attitudes toward heritage participation, based on research on historical building conservation in Hong Kong. In the context of industrial heritage, Ifko (2016) emphasized that highlighting environmental benefits can enhance public support.

According to environmental concern theory, individuals’ environmental attitudes influence their behavior (Dunlap and Van Liere, 1978). When people understand that heritage conservation contributes to environmental sustainability, they are more likely to support and participate in preservation efforts (Zhang and Zhao, 2018). Based on this, the following hypotheses are proposed:

H2a: Environmental sustainability awareness has a significant positive effect on public participation intention.

H2b: Environmental sustainability awareness has a significant positive effect on willingness to pay.

### 3.3 Social identity and public participation

Social identity refers to individuals’ sense of belonging to and identification with a specific social group (Tajfel and Turner, 1979). As a symbol of local history and culture, industrial heritage can strengthen community identity and cohesion (Graham et al., 2000). According to Social Identity Theory, individuals engage in activities that reflect group values to maintain and enhance their identity (Stets and Burke, 2000).

Murzyn-Kupisz and Działek (2013) found that cultural heritage strengthens community identity and fosters participation. Waterton (2005) confirmed the positive impact of social identity on public engagement in industrial heritage preservation in the United Kingdom. In China, Wang and Zan (2011) noted that industrial heritage, as a carrier of urban memory, increases residents’ sense of place and willingness to participate.

Social identity may also influence economic decision-making (Akerlof and Kranton, 2000). When industrial heritage is seen as integral to community identity, individuals are more likely to support it financially (Noonan, 2003). Based on this, the following hypotheses are proposed:

H3a: Social identity has a significant positive effect on public participation intention.

H3b: Social identity has a significant positive effect on willingness to pay.

### 3.4 Perceived government support and public participation

Perceived government support refers to the public’s evaluation of government actions in policy, funding, and management related to industrial heritage conservation (Su and Wall, 2014). As the primary actor in public affairs, the government plays a key role in encouraging public participation (Nasser, 2003).

Nasser (2003) identified effective policies and institutional support as critical to promoting public engagement in heritage conservation. Shipley and Kovacs (2008), in a study of historic building protection in Canada, found that perceived government support positively influences public attitudes. In China, Su et al. (2016) emphasized that government leadership significantly affects public willingness to participate.

According to institutional trust theory, when the public perceives strong and effective government management, they are more likely to engage in related activities (Hetherington, 2004). Moreover, public willingness to pay may increase when the government is seen as actively supporting heritage protection (Throsby and Zednik, 2014). The following hypotheses are proposed:

H4a: Perceived government support has a significant positive effect on public participation intention.

H4b: Perceived government support has a significant positive effect on willingness to pay.

### 3.5 The mediating role of place attachment

Place attachment, defined as the emotional bond between individuals and specific places, may mediate the relationship between perceived value, environmental sustainability awareness, social identity, perceived government support, and both public participation and willingness to pay (Ramkissoon et al., 2013).

First, perceived value of industrial heritage can strengthen place attachment. When individuals recognize its historical, cultural, and educational value, they are more likely to develop emotional connections (Prentice, 2001). Prentice (2001) showed that cultural value perception enhances place attachment among visitors. Environmental sustainability awareness can also influence attachment, as environmental quality is a key component of place bonding (Vaske and Kobrin, 2001).

Second, social identity is closely related to place attachment. Gu and Ryan (2008) found that community identity enhances residents’ attachment to historic neighborhoods. Perceived government support may also indirectly increase place attachment by improving environmental conditions and management quality (Devine-Wright, 2009).

Finally, place attachment can affect both participation and willingness to pay. Ramkissoon et al. (2013) reported that place attachment positively influences pro-environmental behavior among tourists. López-Mosquera and Sánchez (2013) identified it as a key determinant of willingness to pay. The following hypotheses are proposed:

H5a: Place attachment mediates the relationship between perceived value of industrial heritage and public participation intention.

H5b: Place attachment mediates the relationship between perceived value of industrial heritage and willingness to pay.

H6a: Place attachment mediates the relationship between environmental sustainability awareness and public participation intention.

H6b: Place attachment mediates the relationship between environmental sustainability awareness and willingness to pay.

H7a: Place attachment mediates the relationship between social identity and public participation intention.

H7b: Place attachment mediates the relationship between social identity and willingness to pay.

H8a: Place attachment mediates the relationship between perceived government support and public participation intention.

H8b: Place attachment mediates the relationship between perceived government support and willingness to pay.

## 4 Research methods

### 4.1 Research site

**Figure 1:**
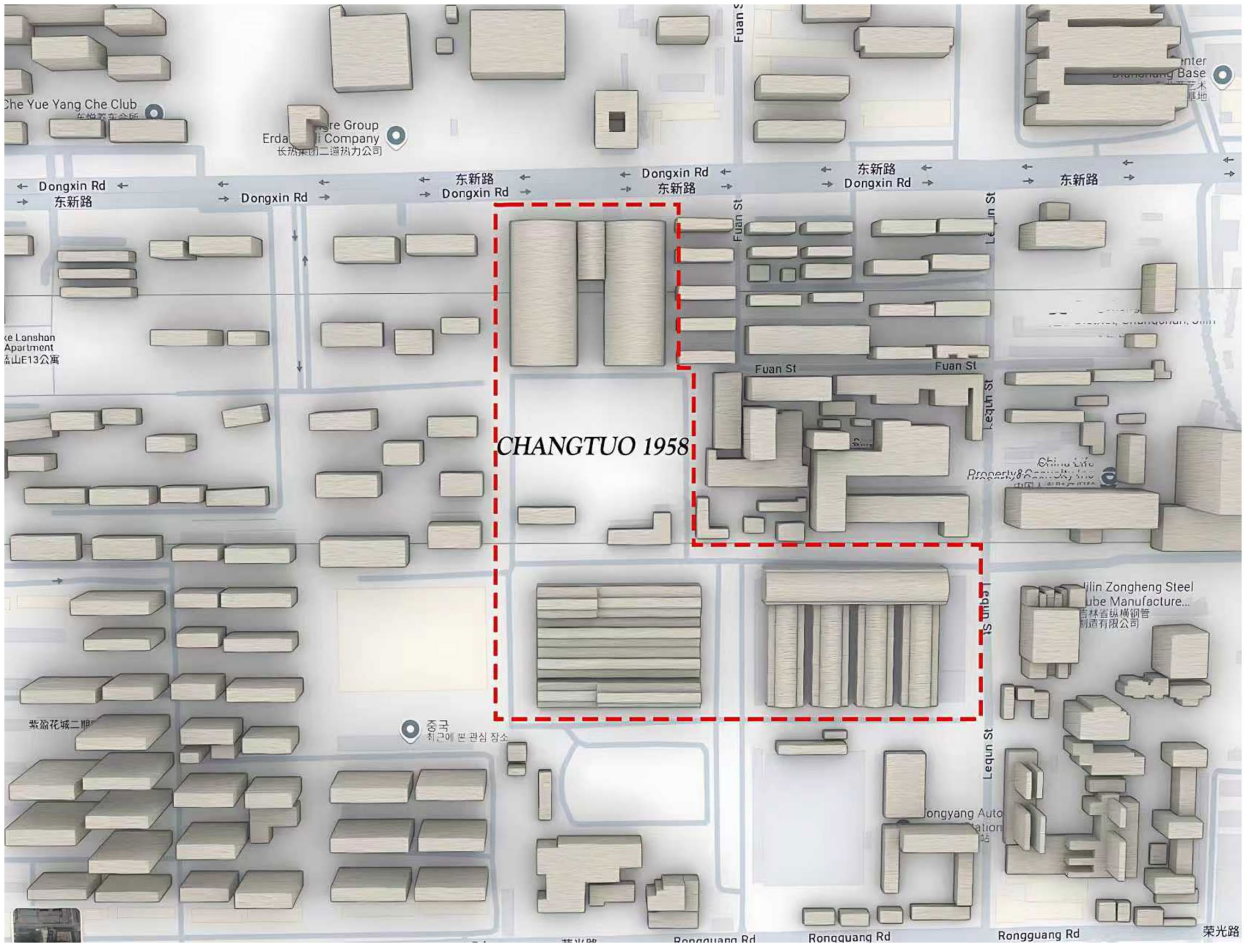
*Changtuo 1958* architectural area spatial distribution map. Source: author.

The study focuses on the “*Changtuo 1958*” industrial heritage site in Changchun, Jilin Province. This site originated as the Changchun Tractor Factory, established on May 29, 1958. It was one of the largest wheel-tractor manufacturing bases in China and a symbol of early industrialization. In the 1980s, its small four-wheel tractors received national awards and even won an international gold medal in Poland. However, due to market changes and industrial restructuring, the factory went bankrupt in 2006. To preserve industrial memory, the site was redeveloped in 2021 as “*Changtuo 1958*” It retained original red-brick buildings and relics, as shown in Figure 1. The old industrial base from 1958 has been transformed into a new urban landmark integrating culture, art, and commerce. Some areas have become creative industry zones, museums, and cultural venues. This case represents a successful example of industrial heritage conservation and sustainable urban regeneration.

### 4.2 Questionnaire design and variable measurement

This study used a structured questionnaire to collect data, consisting of two sections: demographic characteristics and primary research variables. All main constructs were measured using a 5-point Likert scale (1 = strongly disagree, 5 = strongly agree).

Perceived Value of Industrial Heritage (HVP) was measured with five items adapted from Yung and Chan (2012), Xie (2015a), and Xie (2015b), capturing public perceptions of the historical, cultural, educational, esthetic, and economic value of industrial heritage. The Environmental Sustainability Perception (ESP) scale, based on Bullen and Love (2010) and included five items to assess how the public perceives the relationship between industrial heritage conservation and environmental sustainability.

The Social Identity (SI) scale referred to Gu and Ryan (2008) and Wang and Zan (2011), comprising five items to measure the degree to which individuals associate industrial heritage with community identity and belonging. The Perceived Government Support (GSP) scale, adapted from Nasser (2003) and included five items evaluating public perceptions of government policy, funding, and management support for industrial heritage preservation.

Place Attachment (PA) was measured with five items adapted from Williams and Vaske (2003) and Ramkissoon et al. (2013), reflecting emotional connections between individuals and the industrial heritage site. The Public Participation Willingness (PPW) scale was adapted from Hou and Du (2020) and Mydland and Grahn (2012), including five items to assess willingness to participate in heritage conservation activities. The Willingness to Pay (WTP) scale, drawn from Tuan and Navrud (2008) and Chen and Hua (2015), consisted of five items measuring respondents’ intent to provide financial support for industrial heritage conservation.

To ensure contextual relevance, all scale items were translated into Chinese and reviewed by two domain experts from China with backgrounds in heritage studies and environmental psychology. They evaluated the semantic clarity and cultural appropriateness of the items, particularly for constructs such as social identity, place attachment, and perceived government support. Their feedback helped ensure that the measures were suitably adapted to the Chinese sociocultural and institutional context.

### 4.3 Data collection and sample characteristics

Data were collected through a combination of online and offline methods. Offline surveys were randomly distributed at the industrial heritage site, while online surveys were shared through social media platforms and online survey tools targeting local residents. The survey was conducted from March to May 2025. A total of 412 responses were collected, of which 385 were valid after excluding incomplete or invalid questionnaires, resulting in a valid response rate of 93.4%.

The sample included 47.0% of the respondents were male and 53.0% were female. In terms of age, 23.6% were between 18 and 25 years old, 35.8% between 26 and 35, 22.3% between 36 and 45, 12.5% between 46 and 55, and 5.8% were over 56. Regarding education, 3.6% had a junior high school education or below, 12.7% completed high school or vocational school, 21.6% held an associate degree, 44.2% had a bachelor’s degree, and 17.9% held a master’s degree or above. In terms of monthly income, 10.4% earned less than 3,000 RMB, 18.7% earned between 3,001 and 5,000 RMB, 31.2% between 5,001 and 8,000 RMB, 27.5% between 8,001 and 12,000 RMB, and 12.2% above 12,000 RMB. Regarding residence, 36.6% lived near the industrial heritage site, 39.7% were from other areas in the same city, and 23.7% were out-of-town visitors. The variable characteristics presented here are further analyzed in detail in Section 5.2 (Table 1).

**Table 1:** Sample profile.

Variable	*N*	Mean	Std. deviation	Min	Max
Gender	385	1.53	0.50	1	2
Age	385	35.27	10.86	18	65
Education level	385	3.65	0.97	1	5
Monthly income	385	3.12	1.14	1	5
Place of residence	385	1.87	0.78	1	3
Perceived value of industrial heritage	385	4.12	0.68	2.2	5
Environmental sustainability awareness	385	3.95	0.72	1.8	5
Social identity	385	4.03	0.75	1.6	5
Perceived government support	385	3.56	0.89	1	5
Place attachment	385	3.87	0.81	1.4	5
Public participation intention	385	3.72	0.83	1.2	5
Willingness to pay	385	3.41	0.92	1	5

### 4.4 Data analysis method

This study employed SPSS 26.0 and AMOS 24.0 for data analysis. The specific analytical procedures included: (1) descriptive statistical analysis to understand the basic characteristics of each variable; (2) reliability analysis to assess the internal consistency of the questionnaire; (3) correlation analysis to preliminarily examine the relationships among variables; (4) multiple regression analysis to test the effects of independent variables on dependent variables; (5) mediation analysis to examine the mediating role of place attachment; and (6) structural equation modeling (SEM) to comprehensively test the research hypotheses.

## 5 Data analysis and results

### 5.1 Reliability and validity analysis

To assess the reliability and validity of the questionnaire, both reliability and validity analyses were conducted. As shown in Tables 2–5, all Cronbach’s *α* coefficients for the variables exceeded 0.85, indicating strong internal consistency of the measurement scales.

**Table 2:** Reliability analysis.

Variable	Cronbach’s α
Perceived value of industrial heritage	0.876
Environmental sustainability awareness	0.853
Social identity	0.892
Perceived government support	0.865
Place attachment	0.908
Public participation intention	0.882
Willingness to pay	0.871

**Table 3:** Reliability analysis.

Construct	Item	Standardized factor loading	T-value	CR	AVE
Perceived value of industrial heritage (HVP)	HVP1	0.846	20.73***	0.915	0.683
	HVP2	0.852	21.06***		
	HVP3	0.828	19.84***		
	HVP4	0.795	18.42***		
	HVP5	0.810	19.15***		
Perception of environmental sustainability (ESP)	ESP1	0.782	17.93***	0.897	0.635
	ESP2	0.803	18.76***		
	ESP3	0.825	19.53***		
	ESP4	0.798	18.57***		
	ESP5	0.773	17.62***		
Sense of social identity (SI)	SI1	0.841	20.57***	0.923	0.706
	SI2	0.865	21.52***		
	SI3	0.832	20.18***		
	SI4	0.847	20.79***		
	SI5	0.817	19.63***		
Perceived government support (GSP)	GSP1	0.791	18.24***	0.906	0.659
	GSP2	0.836	20.13***		
	GSP3	0.842	20.36***		
	GSP4	0.809	19.05***		
	GSP5	0.775	17.68***		
Place attachment (PA)	PA1	0.862	21.43***	0.934	0.739
	PA2	0.876	22.05***		
	PA3	0.883	22.31***		
	PA4	0.828	20.01***		
	PA5	0.847	20.76***		
Public participation willingness (PPW)	PPW1	0.821	19.74***	0.917	0.689
	PPW2	0.838	20.36***		
	PPW3	0.795	18.63***		
	PPW4	0.854	21.03***		
	PPW5	0.845	20.65***		
Willingness to pay (WTP)	WTP1	0.832	20.04***	0.908	0.665
	WTP2	0.849	20.83***		
	WTP3	0.817	19.52***		
	WTP4	0.773	17.69***		
	WTP5	0.808	19.15***		

***p < 0.001. CR, Composite reliability; AVE, Average variance extracted.

**Table 4:** Distinguishing validity analysis.

Variable	HVP	ESP	SI	GSP	PA
HVP	0.827				
ESP	0.583	0.797			
SI	0.612	0.547	0.84		
GSP	0.432	0.405	0.463	0.812	
PA	0.587	0.523	0.675	0.492	0.86
PPW	0.543	0.518	0.592	0.465	0.628
WTP	0.487	0.462	0.523	0.438	0.572

The bold values on the diagonal represent the square roots of the AVE, while the off-diagonal values indicate the correlation coefficients between variables.

**Table 5:** Credit efficiency simulation fitting.

Fit index	Value	Recommended criterion	Assessment
*χ*^2^/df	2.36	< 3.00	Good
RMSEA	0.059	< 0.08	Good
CFI	0.942	> 0.90	Good
TLI	0.935	> 0.90	Good
SRMR	0.047	< 0.08	Good
GFI	0.912	> 0.90	Good
AGFI	0.895	> 0.80	Good
NFI	0.921	> 0.90	Good
IFI	0.943	> 0.90	Good

RMSEA, Root Mean Square Error of Approximation; CFI, Comparative Fit Index; TLI, Tucker-Lewis Index; SRMR, Standardized Root Mean Square Residual; GFI, Goodness-of-Fit Index; AGFI, Adjusted Goodness-of-Fit Index; NFI, Normed Fit Index; IFI, Incremental Fit Index.

In terms of validity, confirmatory factor analysis was conducted to assess the structural validity of the questionnaire. The results indicated a good model fit: χ^2^/df = 2.36, RMSEA = 0.059, CFI = 0.942, TLI = 0.935, and SRMR = 0.047. All standardized factor loadings exceeded 0.70 and were statistically significant at the *p* < 0.001 level, indicating strong convergent validity. The average variance extracted (AVE) values for all constructs were above 0.50 and greater than the squared correlations between constructs, supporting the discriminant validity of the questionnaire.

Based on the results presented in Tables 3–5, the measurement model in this study demonstrates good validity:

Convergent validity: All standardized factor loadings are greater than 0.70 and statistically significant at the *p* < 0.001 level. The composite reliability (CR) values for all constructs exceed 0.80, and the average variance extracted (AVE) values are all above 0.60, indicating strong convergent validity.

Discriminant validity: As shown in Table 4, the square roots of the AVE values (bolded on the diagonal) are all greater than the correlations between constructs (off-diagonal elements in the same rows and columns), supporting the discriminant validity of the measurement model.

Model fit: As shown in Table 5, all model fit indices meet or approach recommended thresholds, indicating that the measurement model fits the data well.

### 5.2 Descriptive statistics and correlation analysis

Table 1 presents the descriptive statistics for each variable. The mean score for Perceived Value of Industrial Heritage (HVP) is 4.12, indicating a high level of public recognition of the value of industrial heritage. The mean for Environmental Sustainability Perception (ESP) is 3.95, suggesting that the public holds a relatively strong understanding of the relationship between industrial heritage preservation and environmental sustainability. The mean score for Social Identity (SI) is 4.03, reflecting a high level of public agreement on the connection between industrial heritage and community identity.

The mean for Perceived Government Support (GSP) is 3.56, which is relatively lower, indicating that the public perceives governmental support for industrial heritage preservation as insufficient. The mean for Place Attachment (PA) is 3.87, suggesting that the public has a certain emotional connection to industrial heritage sites. The mean for Public Participation Willingness (PPW) is 3.72, indicating a relatively strong willingness among the public to engage in heritage preservation activities. Finally, the mean for Willingness to Pay (WTP) is 3.41, which is comparatively lower, reflecting limited public willingness to provide financial support for industrial heritage preservation.

Table 6 presents the results of the correlation analysis among all variables. Significant positive correlations were found between all variables (*p* < 0.05). The highest correlation coefficient was observed between Perceived Value of Industrial Heritage (HVP) and Social Identity (SI) (*r* = 0.612), indicating a strong relationship between the two. A similarly strong correlation was found between Social Identity and Place Attachment (PA) (*r* = 0.675), supporting their close theoretical association. The correlation between Place Attachment and Public Participation Willingness (PPW) was 0.628, and with Willingness to Pay (WTP) it was 0.572, providing preliminary support for the hypothesis that place attachment may serve as a mediating variable between the independent and dependent variables.

**Table 6:** Relevance analysis.

Variable	HVP	ESP	SI	GSP	PA
HVP	1				
ESP	0.583*	1			
SI	0.612*	0.547*	1		
GSP	0.432*	0.405*	0.463*	1	
PA	0.587*	0.523*	0.675*	0.492*	1
PPW	0.543*	0.518*	0.592*	0.465*	0.628*
WTP	0.487*	0.462*	0.523*	0.438*	0.572*

### 5.3 Regression analysis results

#### 5.3.1 Effects on public participation willingness

Table 7 displays the regression analysis results regarding the impact of independent variables on Public Participation Willingness (PPW). The model demonstrates a good overall fit (*R*^2^ = 0.478, *F* = 38.24, *p* < 0.001). The results show that Perceived Value of Industrial Heritage (HVP) (*β* = 0.215, *p* < 0.01), Environmental Sustainability Perception (ESP) (*β* = 0.187, *p* < 0.01), Social Identity (SI) (*β* = 0.263, *p* < 0.001), and Perceived Government Support (GSP) (*β* = 0.142, *p* < 0.01) all have significant positive effects on public willingness to participate, supporting hypotheses H1a, H2a, H3a, and H4a. Among these, Social Identity has the strongest effect, suggesting that enhancing the public’s social identification with industrial heritage is key to increasing their willingness to participate.

**Table 7:** Regression analysis: public participation willingness.

Variable	Coefficient	Std. error	T-value	P > t	95% Confidence interval
Perceived value of industrial heritage	0.215	0.062	3.47	0.001	[0.093, 0.337]
Perceived environmental sustainability	0.187	0.056	3.34	0.001	[0.077, 0.297]
Sense of social identity	0.263	0.060	4.38	0.000	[0.145, 0.381]
Perceived government support	0.142	0.043	3.30	0.001	[0.057, 0.227]
Gender	−0.023	0.069	−0.33	0.739	[−0.159, 0.113]
Age	0.005	0.003	1.67	0.097	[−0.001, 0.011]
Education level	0.086	0.038	2.26	0.024	[0.011, 0.161]
Monthly income	0.052	0.032	1.63	0.105	[−0.011, 0.115]
Place of residence	−0.097	0.046	−2.11	0.036	[−0.188, −0.006]
Constant	0.487	0.295	1.65	0.100	[−0.093, 1.067]

In terms of control variables, education level (*β* = 0.086, *p* < 0.05) has a significant positive effect on public participation willingness, indicating that higher education levels are associated with stronger willingness to participate. Place of residence (*β* = −0.097, *p* < 0.05) has a significant negative effect, suggesting that individuals living closer to the industrial heritage site exhibit stronger willingness to participate.

#### 5.3.2 Effects on willingness to pay

Table 8 presents the regression analysis results for the effects of independent variables on Willingness to Pay (WTP). The model shows good overall fit (*R*^2^ = 0.423, *F* = 30.56, *p* < 0.001). The results indicate that Perceived Value of Industrial Heritage (HVP) (*β* = 0.183, *p* < 0.05), Environmental Sustainability Perception (ESP) (*β* = 0.162, *p* < 0.05), Social Identity (SI) (*β* = 0.225, *p* < 0.01), and Perceived Government Support (GSP) (*β* = 0.156, *p* < 0.01) all have significant positive impacts on willingness to pay, supporting hypotheses H1b, H2b, H3b, and H4b. Similar to public participation willingness, Social Identity has the strongest influence on willingness to pay.

**Table 8:** Regression analysis: willingness to pay.

Variable	Coefficient	Std. error	T-value	P > t	95% Confidence interval
Perceived value of industrial heritage	0.183	0.071	2.58	0.010	[0.044, 0.322]
Perceived environmental sustainability	0.162	0.064	2.53	0.012	[0.036, 0.288]
Sense of social identity	0.225	0.069	3.26	0.001	[0.089, 0.361]
Perceived government support	0.156	0.049	3.18	0.002	[0.059, 0.253]
Gender	0.042	0.079	0.53	0.596	[−0.114, 0.198]
Age	−0.003	0.004	−0.75	0.453	[−0.010, 0.004]
Education LEVEL	0.112	0.044	2.55	0.011	[0.026, 0.198]
Monthly Income	0.138	0.037	3.73	0.000	[0.065, 0.211]
Place of residence	−0.115	0.053	−2.17	0.031	[−0.219, −0.011]
Constant	0.324	0.338	0.96	0.339	[−0.341, 0.989]

Regarding control variables, education level (*β* = 0.112, *p* < 0.05) and monthly income (*β* = 0.138, *p* < 0.001) show significant positive effects on willingness to pay, indicating that individuals with higher education and income levels are more willing to contribute financially. Place of residence (*β* = −0.115, *p* < 0.05) has a significant negative effect, suggesting that those living closer to the industrial heritage site exhibit stronger willingness to pay.

### 5.4 Mediation effect analysis

To test the mediating role of place attachment, this study adopts the mediation test procedure proposed by Baron and Kenny (1986), as well as the bootstrap method for further verification. Table 9 presents the results of the mediation effect analysis.

**Table 9:** Mediating effect.

Path	Direct effect	Indirect effect	Total effect	Sobel test Z-value	*p*-value
HVP → PA → PPW	0.147*	0.068*	0.215*	3.62	0.00
ESP → PA → PPW	0.128*	0.059*	0.187*	3.21	0.001
SI → PA → PPW	0.156*	0.107*	0.263*	4.53	0.00
GSP → PA → PPW	0.097*	0.045*	0.142*	2.98	0.003
HVP → PA → WTP	0.124*	0.059*	0.183*	3.28	0.001
ESP → PA → WTP	0.112*	0.050*	0.162*	2.89	0.004
SI → PA → WTP	0.134*	0.091*	0.225*	3.87	0.00
GSP → PA → WTP	0.118*	0.038*	0.156*	2.65	0.008

The results indicate that place attachment plays a partial mediating role in the relationships between: Perceived value of industrial heritage and public participation willingness (indirect effect = 0.068, *p* < 0.001), Perceived value of industrial heritage and willingness to pay (indirect effect = 0.059, *p* < 0.01),supporting H5a and H5b.

It also partially mediates the relationships between: Environmental sustainability perception and public participation willingness (indirect effect = 0.059, *p* < 0.01), Environmental sustainability perception and willingness to pay (indirect effect = 0.050, *p* < 0.01),supporting H6a and H6b. Furthermore, place attachment partially mediates: The relationship between social identity and public participation willingness (indirect effect = 0.107, *p* < 0.001).

The relationship between social identity and willingness to pay (indirect effect = 0.091, *p* < 0.001),supporting H7a and H7b. Similarly, it partially mediates: The relationship between perceived government support and public participation willingness (indirect effect = 0.045, *p* < 0.01), The relationship between perceived government support and willingness to pay (indirect effect = 0.038, *p* < 0.01),supporting H8a and H8b.

Among all the mediation paths, social identity has the largest indirect effect on both public participation willingness and willingness to pay through place attachment. This suggests that enhancing social identity can significantly increase individuals’ participation and financial support for industrial heritage protection by strengthening their emotional attachment to the site.

### 5.5 Structural equation modeling analysis

To comprehensively validate the research hypotheses, a structural equation model (SEM) was constructed. The model shows a good fit to the data, with fit indices as follows:

*χ*^2^/df = 2.36, RMSEA = 0.059, CFI = 0.942, TLI = 0.935, SRMR = 0.047 (see Table 10).

**Table 10:** Analysis of structural equation models.

Index	Value	Reference criteria
χ^2^/df	2.36	<3
RMSEA	0.059	<0.08
CFI	0.942	>0.90
TLI	0.935	>0.90
SRMR	0.047	<0.08
χ^2^/df	2.36	<3
RMSEA	0.059	<0.08
CFI	0.942	>0.90

The results of the structural equation model analysis are consistent with those of the regression and mediation analyses, further validating the research hypotheses. Specifically, perceived value of industrial heritage, recognition of environmental sustainability, social identity, and perceived government support all exert significant positive effects on place attachment, public participation intention, and willingness to pay. Place attachment plays a partial mediating role between the independent and dependent variables.

**Figure 2:**
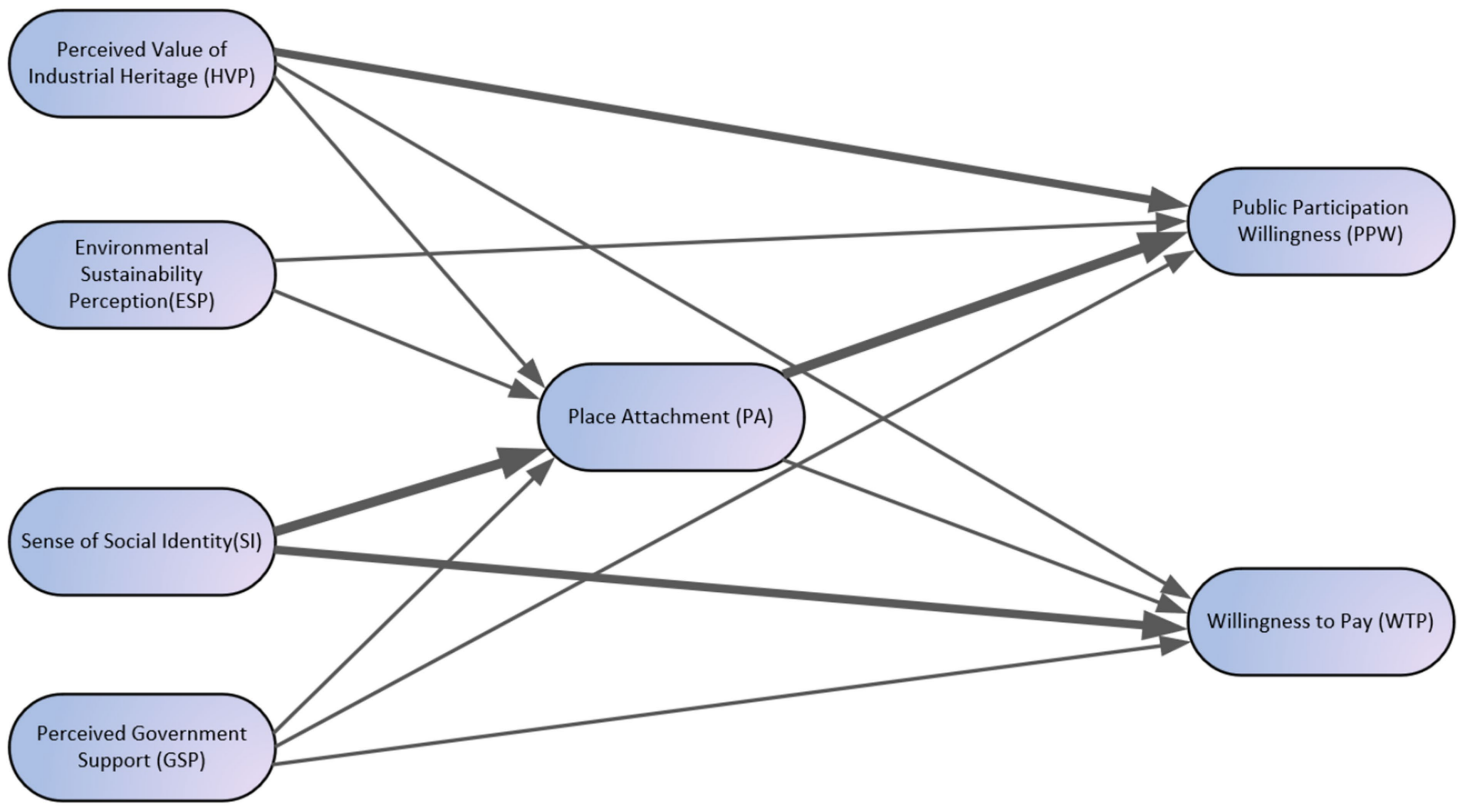
The proposed structural model. Source: author.

Finally, this study generated a framework diagram with direction and intensity based on theoretical assumptions and result analysis (see Figure 2). The model includes four independent variables perceived heritage value, environmental sustainability awareness, social identity, and perceived government support two dependent variables willingness to participate and willingness to pay and one mediating variable, place attachment.

## 6 Discussion

### 6.1 The impact of perceived value of industrial heritage

The findings indicate that the perceived value of industrial heritage has a significant positive impact on public participation intention and willingness to pay, aligning with existing research (Poria et al., 2013; Ung and Vong, 2010). When the public recognizes the historical, cultural, educational, esthetic, and economic value of industrial heritage, they are more likely to be motivated to participate in its preservation and provide financial support. This underscores the importance of enhancing public awareness of the value of industrial heritage.

Compared to traditional cultural heritage, the value of industrial heritage is often less intuitive and harder to grasp (Xie, 2006). Therefore, education, publicity, and interpretive systems are essential in helping the public understand its multifaceted value. It is especially important to highlight its historical value as evidence of industrial civilization and its cultural value as a carrier of urban memory to raise awareness of preservation. Moreover, presenting the economic and social benefits of heritage reuse from a pragmatic perspective may further motivate public participation (Yung and Chan, 2012).

This study also finds that perceived value of industrial heritage indirectly influences public participation and payment intentions through place attachment. This suggests that recognizing the value of industrial heritage not only directly affects willingness to participate but also strengthens emotional bonds with heritage sites, thereby further increasing support (Ramkissoon et al., 2013). This finding enriches the theoretical understanding of how perceived value functions by emphasizing the role of emotional connection.

### 6.2 The impact of environmental sustainability recognition

Results show that recognition of environmental sustainability has a significant positive impact on public participation and willingness to pay, expanding prior research (Bullen and Love, 2010; Yung and Chan, 2012). When people understand the connection between industrial heritage preservation and sustainable development, they are more likely to support such efforts. Framing industrial heritage conservation within a sustainability narrative can thus enhance public endorsement.

In the post-industrial era, environmental awareness and sustainability principles are gaining momentum (Dunlap and Van Liere, 1978). As former sources of pollution, industrial heritage sites are closely tied to environmental issues (Loures and Vaz, 2018). Emphasizing how industrial heritage transformation aligns with green building standards, reduces resource waste, and improves energy efficiency can strengthen public recognition and support (Ifko, 2016). In the Chinese context, linking heritage conservation to ecological civilization goals and carbon neutrality targets can foster broader social support.

Moreover, this study finds that environmental sustainability recognition indirectly impacts participation and payment intentions through place attachment, indicating that perceived environmental quality significantly influences emotional bonds with a place (Vaske and Kobrin, 2001). When the public believes that industrial site reuse benefits the environment, they are more likely to develop positive affect and support its conservation.

### 6.3 The impact of social identity

Social identity exerts the strongest influence on participation and payment intentions, echoing earlier studies (Murzyn-Kupisz and Działek, 2013; Waterton, 2005). When the public views industrial heritage as integral to community identity and local culture, they are more inclined to support and participate in its preservation. This highlights the socio-cultural functions and symbolic significance of industrial heritage.

In China’s rapid urbanization, many cities suffer from homogeneous development (Wang and Zan, 2011). As carriers of distinctive urban memory and cultural identity, industrial heritage plays a vital role in enhancing city character and resident identity (Xie, 2015a; Xie, 2015b). By integrating local features and historical narratives into cultural and community-building efforts, social identity can be reinforced, promoting greater public participation (Wang et al., 2020).

This study also finds that social identity has the largest indirect effect on participation and willingness to pay via place attachment, indicating a close relationship between the two. This finding contributes to theoretical development by demonstrating how social and emotional mechanisms jointly influence public involvement (Gu and Ryan, 2008).

### 6.4 The impact of perceived government support

Perceived government support has a significant positive effect on public participation and payment intentions, consistent with prior research (Nasser, 2003; Shipley and Kovacs, 2008). When the public perceives strong governmental commitment and effective management, they are more willing to engage and provide support. This underlines the guiding and exemplary role of government in heritage preservation.

In China’s institutional context, the government plays a leading role in public affairs (Su and Wall, 2014). Its stance and actions toward industrial heritage conservation directly affect public behavior (Su et al., 2016). Formulating sound policies, providing financial support, and creating participatory mechanisms can effectively increase public involvement (Li et al., 2019).

Notably, the average score for perceived government support (3.56) is relatively low, suggesting a need for improved communication and policy visibility. Governments should strengthen public outreach and engagement, enhance transparency, and foster multi-stakeholder collaboration in heritage conservation efforts (Hou and Du, 2020).

### 6.5 The mediating role of place attachment

The study confirms that place attachment partially mediates the relationship between perceived factors and public willingness, advancing theoretical understanding in industrial heritage research. As an emotional bond between individuals and places, place attachment is a key psychological mechanism influencing participatory behavior (Ramkissoon et al., 2013; Vong, 2015).

First, perceived value, environmental recognition, social identity, and government support all enhance place attachment. When individuals perceive value, environmental benefits, social significance, and institutional backing, they are more likely to form emotional ties with heritage sites (Prentice, 2001).

Second, place attachment significantly increases participation and payment intentions. Emotional connections encourage people to invest time, energy, and financial resources in preservation efforts (López-Mosquera and Sánchez, 2013). Strategies such as organizing community events, sharing industrial stories, and collecting oral histories are essential in nurturing these bonds (Wheeler, 2014).

This perspective echoes recent work emphasizing that heritage-related emotional and identity-based mechanisms are central to subjective well-being outcomes (Kong et al., 2024).

## 7 Conclusion and implications

### 7.1 Theoretical contributions

The present study elucidates the psychological mechanisms underpinning public support for industrial heritage conservation, with a specific focus on the mediating role of place attachment. First, findings confirm that perceived value of industrial heritage significantly enhances both willingness to participate in conservation activities and willingness to provide financial support. This aligns with theoretical expectations derived from the Theory of Planned Behavior and Value–Attitude–Behavior frameworks, which posit that positive evaluations of expected outcomes drive behavioral intentions. In practical terms, these results underscore the importance of interpretive initiatives and educational programs that clearly communicate the multifaceted significance of industrial heritage, including its historical, cultural, and economic dimensions.

Second, environmental sustainability awareness emerged as a robust predictor of conservation intentions. Respondents who recognized the environmental benefits of adaptive reuse such as reduced resource consumption and lowered carbon emissions demonstrated stronger engagement and financial commitment. This finding extends prior research in heritage tourism by integrating sustainability considerations into the value construct and highlights the efficacy of framing industrial relics within ecological narratives to foster public endorsement.

Third, social identity exerted the most pronounced effect on both participation and payment intentions. Consistent with Social Identity Theory, individuals who perceived industrial heritage as integral to community cohesion and the collective memory displayed heightened motivation to support its preservation. Furthermore, social identity exhibited the largest indirect effect on behavioral outcomes through place attachment, indicating that identity-based perceptions translate into emotional bonds that, in turn, drive pro-conservation behaviors. This interplay underscores the dual role of heritage as a symbolic anchor of local identity and an emotional touchstone.

Fourth, perceived government support positively influenced conservation intentions, albeit to a lesser extent than social identity. The results suggest that visible and credible institutional backing manifested through policy incentives, funding mechanisms, and transparent management can strengthen public trust and encourage active engagement. Notably, the relatively modest mean score for government support indicates an opportunity for policymakers to improve communication and co-management frameworks to elevate public perceptions.

Finally, place attachment was confirmed as a partial mediator linking cognitive and social drivers to conservation intentions. Each predictor perceived value, environmental awareness, social identity, and government support contributed to stronger emotional bonds with the heritage site, which in turn reinforced both participation and financial support. This mediator underscores the central role of affective commitment in transforming abstract evaluations into concrete actions, and suggests that heritage managers should prioritize experiential and immersive approaches such as community storytelling, interactive tours, and participatory events to cultivate deeper place attachment.

### 7.2 Practical implications

Enhance interpretive communication to convey the comprehensive value of industrial heritage. Multi-modal platforms including digital media, on-site exhibits, and guided narratives should emphasize historical achievements, cultural legacies, and economic revitalization potentials.

Integrate sustainability messaging into heritage programming. Demonstrating the environmental benefits of adaptive reuse, and aligning conservation efforts with broader ecological and carbon-neutrality goals, can galvanize support from environmentally conscious stakeholders.

Strengthen community identity through culturally resonant activities. Hosting local exhibitions, festivals, and workshops in heritage venues can reinforce the collective memory, foster pride, and stimulate social cohesion.

Bolster institutional credibility by establishing transparent governance and multi-stakeholder collaboration. Providing clear policy guidelines, accessible funding channels, and participatory decision-making platforms will enhance public trust and commitment.

Foster emotional engagement via immersive experiences. Initiatives such as oral history projects, interactive site reconstructions, and volunteer programs can deepen place attachment and transform visitors into advocates.

It is also important to recognize that heritage conservation often necessitates context-specific and culturally sensitive approaches. International organizations such as UNESCO and ICOMOS have long emphasized the importance of tailoring conservation strategies to the unique historical, social, and environmental characteristics of each site. Rather than pursuing generalized models, effective heritage management should be guided by localized needs, stakeholder values, and institutional frameworks. This perspective reinforces the need for adaptive policies that align with global best practices while respecting local particularities.

### 7.3 Limitations and future research

While this study offers valuable insights into the psychological mechanisms driving public support for industrial heritage conservation, several limitations must be acknowledged. First, the research is based on cross-sectional data collected from a single case site, which limits the generalizability of the findings. Future studies could employ longitudinal or multi-site comparative designs to capture temporal dynamics and regional differences in public engagement behavior.

Second, although this study incorporates both online and offline data collection to enhance sample diversity and reach, the mixed-mode approach may have introduced sampling bias, including self-selection effects and geographic overrepresentation. Notably, 36.6% of respondents were residents living near the heritage site, whose perceptions and emotional bonds may differ substantially from those of non-local visitors. While the current study did not explicitly distinguish between locals and tourists in the analysis, this distinction may be important—visitor status or spatial proximity could serve as potential moderating variables influencing the effects of place attachment, identity, and perceived value on conservation intentions. Future research is encouraged to explore these differences more systematically.

Third, although established measurement scales were employed, they were originally developed in Western contexts. Despite a careful translation–back translation process and expert review by two domain scholars from China, cultural interpretations of constructs such as social identity, place attachment, and government support may still vary, and further localization of measurement tools would enhance conceptual sensitivity.

Finally, the self-reported nature of the data may be subject to social desirability bias, especially for items related to financial support and participation willingness. Experimental designs or behavioral tracking methods could provide more objective validation of public engagement outcomes in future studies.

### 7.4 Conclusion

By integrating cognitive evaluations, social identity constructs, and emotional attachments, this research advances theoretical understanding of public engagement in industrial heritage conservation. The demonstrated mediating role of place attachment offers a nuanced perspective on how value, sustainability awareness, identity, and institutional perceptions coalesce to shape behavioral intentions. These insights provide a robust foundation for evidence-based heritage management practices aimed at sustainable urban regeneration and community empowerment.

## Data Availability

The raw data supporting the conclusions of this article will be made available by the authors, without undue reservation.
